# Plasma markers of COVID-19 severity: a pilot study

**DOI:** 10.1186/s12931-022-02272-7

**Published:** 2022-12-13

**Authors:** Julia Beimdiek, Sabina Janciauskiene, Sabine Wrenger, Sonja Volland, Adriana Rozy, Jan Fuge, Beata Olejnicka, Isabell Pink, Thomas Illig, Alexander Popov, Joanna Chorostowska, Falk F. R. Buettner, Tobias Welte

**Affiliations:** 1 Department of Pulmonary and Infectious Diseases, Hannover Medical School, BREATH German Center for Lung Research (DZL) Hannover University School, Carl-Neuberg-Str. 1, 30625 Hannover, Germany; 2 Hannover Unified Biobank, Hannover Medical School, Feodor-Lynen-Str. 15, 30625 Hannover, Germany; 3 Department of Genetics and Clinical Immunology, National Institute of Tuberculosis and Lung Diseases, 26 Plocka St., 01138 Warsaw, Poland; 4 Institute of Clinical Biochemistry, Hannover Medical School, Carl-Neuberg-Str. 1, 30625 Hannover, Germany

**Keywords:** COVID-9 severity, Acute phase proteins, Cell death, Inflammation, N-glycosylation, Trombosis

## Abstract

**Background:**

SARS-CoV-2 infected patients show heterogeneous clinical presentations ranging from mild symptoms to severe respiratory failure and death. Consequently, various markers reflect this wide spectrum of disease presentations.

**Methods:**

Our pilot cohort included moderate (n = 10) and severe (n = 10) COVID-19 patients, and 10 healthy controls. We determined plasma levels of nine acute phase proteins (APPs) by nephelometry, and full-length (M65), caspase-cleaved (M30) cytokeratin 18, and ADAMTS13 (a disintegrin-like and metalloprotease with thrombospondin type-1 motif 13) by ELISA. In addition, we examined whole plasma N-glycosylation by capillary gel electrophoresis coupled to laser-induced fluorescence detection (CGE-LIF).

**Results:**

When compared to controls, COVID-19 patients had significantly lower concentrations of ADAMTS13 and albumin (ALB) but higher M30, M65, α1-acid glycoprotein (AGP), α1-antitrypsin (AAT), ceruloplasmin (CP), haptoglobin (HP), and high-sensitivity C-reactive protein (hs-CRP). The concentrations of α1-antichymotrypsin (ACT), α2-macroglobulin (A2MG) and serum amyloid A (SAA) proteins did not differ. We found significantly higher levels of AAT and M65 but lower ALB in severe compared to moderate COVID-19 patients. N-glycan analysis of the serum proteome revealed increased levels of oligomannose- and sialylated di-antennary glycans and decreased non-sialylated di-antennary glycan A2G2 in COVID-19 patients compared to controls.

**Conclusions:**

COVID-19-associated changes in levels and N-glycosylation of specific plasma proteins highlight complexity of inflammatory process and grant further investigations.

## Introduction

The coronavirus disease of 2019 (COVID-19) is caused by severe acute respiratory syndrome coronavirus 2 (SARS-CoV-2). Most infected people experience mild to moderate respiratory symptoms and recover without requiring special care. However, some people get seriously ill or die. In general, older people and people with other medical conditions like obesity, cardiovascular disease, diabetes, chronic respiratory disease, or cancer are more likely to develop serious illness. The wide spectrum of clinical presentations of SARS-CoV-2 infected patients implies the involvement of many biological pathways like immunological, inflammatory, and coagulative, and to those pathways related biomarkers. Accumulated data shows that in response to the SARS-CoV-2 infection global changes occur in protein synthesis, processing and posttranslational modifications, like phosphorylation and glycosylation [1]. Some of these changes might reflect the severity of COVID-19. Hitherto, changes in concentrations of D-dimer, cardiac troponin I, renal biomarkers, such as serum urea, creatinine and markers of glomerular filtration rate, C-reactive protein (CRP), ferritin, and certain cytokines/chemokines may have a diagnostic value [2–4]. Several studies reported that circulating levels of damage associated molecular patterns, such as cytoskeletal keratin-18 (CK18) fragment M30/M65 ratio (an indicator of cell apoptosis in relation to the total cell death), HMGB-1 or mitochondrial DNA may also reflect the severity of COVID-19 patients [5–7]. The activity of ADAMTS13 (a disintegrin and metalloprotease with thrombospondin type 1 motif 13), a regulator of von Willebrand factor (VWF) multimer disruption, typically decline with the increasing extent of inflammatory responses [8]. Recent data show that VWF antigen (VWF:Ag) to ADAMTS13 activity ratio is strongly associated with SARS-CoV-2 severity [9].

High levels of pro-inflammatory cytokines also linked to severity and poor outcomes in SARS-CoV-2 infection [10, 11]. For example, the levels of IL-6 rise sharply in severe manifestations of COVID-19. One meta-analysis reviewing six studies reported that mean IL-6 concentrations are 2.9-fold higher in patients with severe compared to those with non-severe COVID-19 disease [12]. As a matter of fact, IL-6 is the only cytokine that affects liver synthesis and secretion of the full spectrum of acute phase proteins (APPs) [13]. Other inflammatory cytokines, like IL-1β and TNFα, modulate synthesis of specific APPs and can have additive, or inhibitory effects on IL-6-induced APPs expressions [14]. In turn, the synthesis of APPs may be influenced by insulin, dexamethasone, and glucagon [15].

As mentioned above, C-reactive protein (CRP) can be used as a diagnostic marker reflecting the severity and prognosis of COVID-19 [16, 17], and the levels of CRP are helpful to differentiate between viral and bacterial infections [18, 19]. Decrease in serum albumin, which is a negative APP, also suggested as a marker of the severity of SARS-CoV-2 infection [20].

Some studies reveal that the host’s protein glycosylation is altered upon infection with SARS-CoV-2 as well. For example, critically sick patients develop high concentrations of IgG antibodies where the conserved N-glycan in the Fc domain is not fucosylated thereby causing an amplified cytokine storm [21]. The glycosylation pattern of AAT is also modified in severe cases of COVID-19, which affects anti-inflammatory properties of AAT protein [22].

Alterations in human plasma protein levels and/or their molecular forms indicate pathophysiological changes caused by various diseases, including viral infections. Therefore, host proteins might not only provide valuable insights for the pathogenesis of diseases, including COVID-19, but also might be useful as clinical biomarkers, either individually or in combinations, to monitor and evaluate the disease development. To explore the clinical value of plasma inflammatory proteins, we enrolled twenty COVID-19 patients, with moderate or severe symptoms and ten non-COVID-19 controls. We performed whole plasma protein N-glycosylation analysis, and measured plasma levels of M30 and M65, as cell death markers, ADAMTS13, as a marker limiting platelet-rich thrombus formation, and various APPs, including albumin (ALB), α1-acid glycoprotein (AGP), α1-antitrypsin (AAT), ceruloplasmin (CP), haptoglobin (HP), high-sensitivity C-reactive protein (hs-CRP), α2-macroglobulin (A2MG), α1-antichymotrypsin (ACT) and serum amyloid A (SAA). We hoped that in addition to previous reports, results from this pilot study could confirm and/or uncover plasma marker candidates associated with COVID-19 symptom severity.

## Material and methods

### Patients and biomaterial

Plasma samples prepared from ethylenediaminetetraacetic acid (EDTA) blood were obtained from the Hannover Unified Biobank (HUB). The ethics committee of the Hannover Medical School (MHH) (ethics vote 9001_BO_K) approved the sample collection. Sample processing and storage was performed following the standard procedures of HUB as described by Kopfnagel et al. [23]. For analysis, plasma samples from 20 patients were available: 10 from patients with severe COVID-19; and 10 from patients with moderately severe COVID-19 illness. Plasma samples from 10 age matched healthy donors were obtained from HUB biobank, collected in the frame of a healthy cohort set up by different northern German biobanks [24]. For this cohort, decentralized SOP guided recruitment of healthy volunteers without apparent diseases was carried out. Samples were collected between February 2018 and October 2018. According to the inclusion criteria, adult (≥ 18 years) and healthy Caucasian volunteers were included. Healthy controls had no systemic steroid or antibiotic therapy and respiratory tract infection in the last month, no asthma, autoimmune diseases, severe and very severe chronic obstructive pulmonary disease, GOLD (Global initiative for chronic Obstructive Lung Disease, http://www.goldcopd.org, version 2010), diabetes mellitus, pregnancy or lactating, active tuberculosis (current or in the past) and, immunosuppression (acquired, iatrogenic, or congenital).

### Human M30 and M65 ELISA

CK18-Asp396 neo-epitope (M30-Apoptosense) and total soluble CK18 levels in plasma samples were measured by using commercially available ELISA kits, M30-Apoptosense assay kit (Cat# 10011, PEVIVA AB, Bromma, Sweden) and M65-ELISA assay kit (Cat# 10020, PEVIVA AB, Bromma, Sweden) according to the manufacturer’s instructions. Briefly, 25 µl of the sample (standard, blank or serum samples) was added to the assay plate and incubated for 4 h for M30 and 2 h for M65, respectively on a plate shaker at 300 rpm. The unbound conjugate was removed by washing five times, and afterwards 200 µl of TMB (3,3’, 5,5’ tetramethyl-benzidine) solution was added to each well for 20 min. The reaction was terminated by stop solution and absorbance was measured at 450 nm (Tecan Infinite M200, Männedorf, Switzerland).

### Human ADAMTS13 ELISA

Human plasma ADAMTS13 concentrations were determined by using a commercially available ADAMTS13 ELISA kit (Cat# ab2345599, Abcam, Cambridge, UK) according to the manufacturer’s instructions. In brief, 50 µl of sample (standard, blank or diluted serum samples), plus 50 µl of antibody cocktail mixture was added to each well and incubated for 1 h on a plate shaker at 400 rpm. The unbound conjugate was removed by washing three times, and afterwards 100 µl of TMB solution was added for 20 min in the dark. The reaction was terminated by adding stop solution and analyzed at 450 nm by microplate reader (Tecan Infinite M200, Männedorf, Switzerland). Assay sensitivity 16 pg/ml, and detection range 0.125–10 ng/ml.

### Analysis of plasma APPs

Plasma concentrations of APPs were measured blindly using the nephelometric method (IMMAGE 800 Protein Chemistry Analyzer, Beckman Coulter Inc., CA, USA) at the Department of Genetics and Clinical Immunology at the National Institute of Tuberculosis and Lung Diseases, Warsaw. Analysis sensitivity for measured APPs was for ALB: 22.2 mg/dl, AAT: 10 mg/dl, AGP: 35 mg/dl, hs-CRP: 0.02 mg/dl, CP: 2 mg/dl, HP: 5.83 mg/dl, and A2MG: 40 mg/dl. Plasma levels of ACT and SAA were measured by ELISA sandwich kit from BT Lab Bioassay Technology Laboratory (Shanghai, China) at 450 nm in a spectrophotometric reader Infinite M200 (Tecan, Austria). Assay sensitivity for ACT was 5.17 µg/ml and for SAA was 0.024 µg/ml. All plasma samples were analyzed at the same time, to control for testing variability.

### N-glycan analysis by multiplexed capillary gel electrophoresis coupled to laser-induced fluorescence detection (CGE-LIF)

Sample preparation for CGE-LIF N-glycan analysis was performed as described previously [25, 26] with slight modifications. Briefly, N-glycans from whole blood serum were dissolved in SDS (2%, w/v) in phosphate buffer, the remaining SDS was neutralized with IGEPAL CA-630 (8%, v/v, Sigma-Aldrich), and glycoproteins were digested in solution with peptide-N-glycosidase F (PNGaseF from *Elizabethkingia meningoseptica*; BioReagent grade, Sigma-Aldrich) to release attached N-glycans. Released N glycans were fluorescently labeled with 8-aminopyrene-1,3,6-trisulfonic acid (APTS; Sigma-Aldrich). Briefly, the glycans were solved in 2 μl APTS (20 mM in 3.5 M citric acid), 2 μl 2-picoline borane complex (PB, 2 M in DMSO, Merck) as a reducing agent, and 2 μl water. After incubation for 16.5 h at 37 °C in darkness, the excess of APTS and reducing agent were removed by HILIC-SPE. Analysis of labeled N-glycans was conducted by xCGE-LIF using an ABI PRISM 3100-Avant Genetic Analyzer (advanced biolab service GmbH, Munich, Germany) and Run 3100-Avant Data Collection Software v.2.0. N-glycans were injected for 15 s at 1.6 kV and after a data delay of 50 s, data was collected for 45 min at 15 kV and 60 °C. Resulting data was analyzed using the GeneMapperTM Software v.3.7. To enable comparison of N-glycan levels between different samples, relative signal intensities of peaks from xCGE-LIF electropherograms were calculated as a percentage referring to the sum of all peak heights within each sample. N-glycan annotation was performed using an in-house established N-glycan database.

### Statistical analysis

The IBM SPSS Statistics (version 27.0, IBM Corp., Armonk, New York) and STATA 13.0 (StataCorp LP, College Station, Texas, USA) statistical software packages were used to analyze the data. Graphs were prepared with Graphpad Prism V9 (Insight Partners, New York City, New York, USA). Categorical variables are shown as numbers (n) and percentages (%). Continuous variables are shown as median (interquartile range, IQR), unless indicated otherwise. Continuous variables were tested using the Kolmogorov–Smirnov test for normal distribution. For comparisons of patient groups Fisher exact test, chi-square test, Mann–Whitney U-test or paired t-test were used as appropriate. Pearson correlation coefficient was calculated to show correlation between continuous variables. All tests were two-sided, p-values < 0.05 were considered statistically significant. Statistical differences of relative N-glycan levels were assessed by individual comparisons between the control group and moderate or severe COVID-19 groups.

## Results

### Patients and controls

We analyzed plasma APP concentrations in retrospectively collected (between April and September 2020) samples from 20 COVID-19 patients: 10 with moderate and 10 with severe COVID-19 symptoms. According to treatment guidelines, moderate COVID-19 disease includes lower respiratory symptoms with a peripheral oxygen saturation (SpO_2_) equal or higher than 94% on room air, severe disease is defined by SpO_2_ < 94%, more than 30 breaths/min, or lung infiltrates > 50%, and critical illness include respiratory failure, septic shock and/or multiple organ dysfunction [27].

From the patients of the moderate COVID-19 group, 3 patients were admitted to an intensive care unit (ICU) or got temporary ventilation. All patients of the severe COVID-19 group needed intensive care and mechanical ventilation, 3 of them were supported with extracorporeal membrane oxygenation (ECMO). Detailed characteristics of the cohort are presented in Table 1. For a reference, plasma protein analysis was performed in age matched healthy controls.

**Table 1:** Cohort description

Groups	Severe COVID-19	Moderate COVID-19	Healthy controls
Number of individuals (n (%))	10 (33.3)	10 (33.3)	10 (33.3)
Age (median (IQR)	41 (31–63)	64 (44–65)	49 (26–54)
Gender (n (%))			
Female	5 (16.7)	3 (10.0)	5 (16.7)
Male	5 (16.7)	7 (23.3)	5 (16.7)
Comorbidities (Yes/no/unkown, n/n/n)			
Lung disease	1/9/0	0/7/3	0/0/10
Diabetes	1/9/0	1/6/3	0/0/10
Heart disease	1/9/0	2/5/3	0/0/10
Adiposity	4/6/0	3/4/3	0/0/10
Hypertension	2/5/3	2/4/4	0/0/10
Cardiovascular disease	1/6/3	1/5/4	0/0/10
Renal insufficeincy	1/6/3	1/5/4	0/0/10
Depression	1/6/3	1/5/4	0/0/10
Liver disease	1/6/3	0/6/4	0/0/10
Obstructive lung disease	1/6/3	0/6/4	0/0/10
Chronic therapy (Yes/no/unkown, n/n/n)			
Cortisone	2/8/0	0/7/3	0/0/10
Immunosuppressive drugs	2/8/0	0/7/3	0/0/10
Place of birth (Europe/other/unkown, n/n/n)	4/3/3	5/1/4	0/0/10
Smoking status (never-/ex-/unkown, n/n/n)	5/2/3	4/3/3	0/0/10
Day of sampling (disease day, median (IQR))	8.5 (5.8–25.8)	12.0 (7.0–18.0)	–
Acute COVID-19 therapy (n (%))			
ICU	10 (33.3)	3 (10.0)	–
Ventilation	10 (33.3)	5 (16.7)	–
ECMO	3 (10.0)	0 (0.0%)	–

*ECMO* extracorporeal membrane oxygenation, *ICU* intensive care unit, *IQR* interquartile range. Values, which are not available, are called “Unknown”

### Plasma levels of M30 and M65

**Fig. 1:**
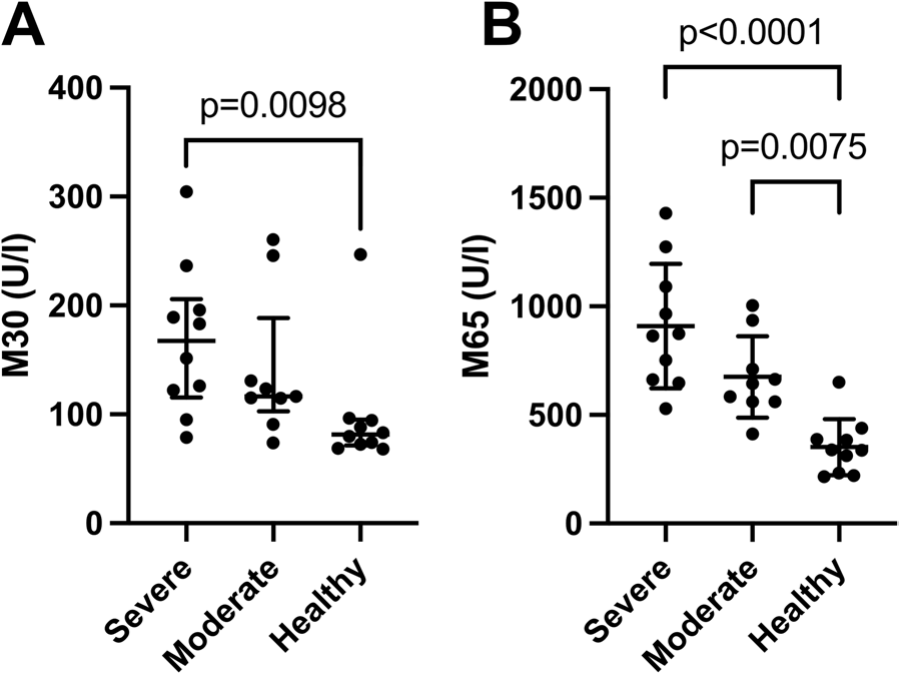
Cell death (M65) and apoptosis (M30) markers in COVID-19 patients with different disease severities: **A** Plasma levels of M30 were measured in 10 severely and 10 moderately ill COVID-19 patients as well as in 10 healthy controls. Values are given as median (IQR). P-values were calculated by Kruskal–Wallis test and Dunn’s multiple comparison test. **B** Plasma levels of M65 were measured in 10 severely and 10 moderately ill COVID-19 patients as well as in 10 healthy controls. Values are given as mean (SD). P-values were calculated by one-way ANOVA test. A p-value below 0.05 was considered significant

Compared with healthy controls, severe COVID-19 patients had significantly higher levels of the apoptosis indicator M30 (Fig. 1A). However, independently on disease severity COVID-19 patients had higher levels of the total cell death indicator M65 than controls (Fig. 1B).

We analysed CK18-M30/M65 ratio, as an indicator of the fraction of cells undergoing apoptosis versus all types of cell death. The M30/M65 ratio did not differ significantly between severe and moderate patients [mean (SD), n = 10, severe: 0.19 (0.07) and moderate: 0.203 (0.04), p = 0.64] but was significantly lower as compared to healthy controls [mean (SD), n = 10, controls: 0.27 (0.06) vs. severe (p = 0.01) and moderate (p = 0.029)].

### Plasma levels of ADAMTS13

**Fig. 2:**
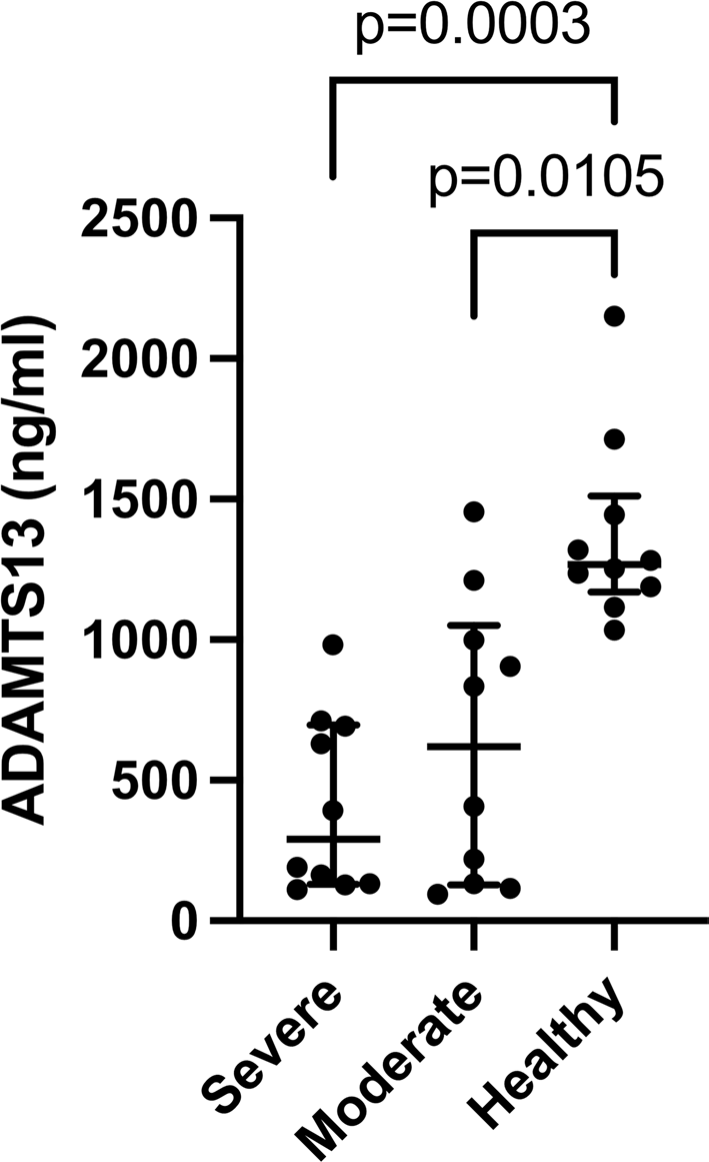
Thrombosis marker ADAMTS13 in COVID-19 patients with different disease severities: Plasma levels of ADAMTS13 were measured in 10 severely and 10 moderately ill COVID-19 patients as well as in 10 healthy controls. Values are given as median (IQR). P-values were calculated by Kruskal–Wallis test and Dunn’s multiple comparison test. A p-value below 0.05 was considered a significant

As illustrated in Fig. 2, patients with mild and severe COVID-19 symptoms had significantly lower plasma levels of ADAMTS13 than healthy controls. Decreased levels of ADAMTS13 were linked with thrombotic events observed in patients with COVID-19 [28].

### Plasma levels of APPs

As presented in Table 2, when compared to controls, COVID-19 patients independently on their disease severity had lower plasma levels of ALB (by 42%) but significantly higher levels of AGP (by 2.4-fold), AAT (by 1.9-fold), CP (by 1.4-fold), HP (by 3.8-fold), and hs-CRP (by 52-fold). Plasma levels of A2MG, ACT and SAA did not differ significantly between COVID-19 patients and controls.

**Table 2:** Acute-phase protein plasma levels in COVID-19 patients compared to healthy controls (mg/dl), except for ACT and SAA (µg/ml)

APP	COVID-19	n	Healthy controls	n	p-value
ACT	1437 (1356–1626)	20	1579 (1484–1856)	9	p = 0.066
SAA	49.33 (45.30–52.81)	20	54.69 (47.43–70.33)	8	p = 0.104
AGP	167.0 (124.5–209.8)	20	68.9 (57.2–82.2)	10	**p < 0.001**
HP	271.5 (189.3–335.8)	20	71.4 (43.5–104.0)	10	**p < 0.001**
AAT	235.5 (180.3–270.0)	20	127.5 (119.3–139.5)	10	**p < 0.001**
hs-CRP	9.680 (4.123–18.925)	20	0.185 (0.063–0.827)	10	**p < 0.001**
A2MG	112.5 (108.0–154.8)	20	128.5 (111.5–200.3)	10	p = 0.243
CP	41.1 (35.7–52.5)	20	28.95 (23.7–35.18)	10	**p = 0.001**
ALB	2540 (1915–3135)	20	4390 (3918–4833)	10	**p < 0.001**

Data presented as median and interquartile range (IQR)

*A2MG* alpha2-macroglobulin, *AAT* alpha1-antitrypsin, *ACT* alpha 1-antichymotrypsin, *AGP* alpha-1 acid glycoprotein, *ALB* albumin, *CP* ceruloplasmin, *hs-CRP* high sensitivity C-reactive protein, *HP* haptoglobin, *SAA* serum amyloid A. Two-sided p-values were calculated with Mann–Whitney-U test, bold highlights statistical significance

Next, we investigated whether plasma APP concentrations differ between COVID-19 patients with different disease severity. As shown in Table 3, except for AAT and ALB, plasma concentrations of other APPs did not differ significantly between patients with moderate and severe COVID-19. Plasma levels of AAT were significantly higher (by 1.4-fold) while ALB lower (by 1.6-fold) in severe patients as compared to those with moderate disease. Albeit without statistical significance, levels of AGP, HP, and CP also increased but A2MG decreased in COVID-19 severity-dependent manner.

**Table 3:** Plasma levels of acute-phase protein in patients with different severity of COVID-19

APP	Moderate COVID-19	Severe COVID-19	p-value
ACT	1459 (1418–1726)	1392 (1309–1557)	p = 0.131
SAA	49.49 (45.33–55.58)	49.33 (44.85–50.56)	p = 0.597
AGP	150.0 (123.0–201.3)	173.5 (131.5–226.5)	p = 0.472
HP	253.0 (195.8–317.5)	271.5 (156.3–365.3)	p = 0.821
AAT	188.5 (150.3–253.3)	267.0 (227.0–327.3)	**p = 0.013**
hs-CRP	8.305 (0.716–13.88)	12.65 (6.883–20.56)	p = 0.226
A2MG	136.5 (106.0–164.0)	109.0 (105.9–126.5)	p = 0.225
CP	39.05 (35.83–46.50)	47.80 (34.90–54.18)	p = 0.273
ALB	3110 (2748–3640)	1930 (1825–2278)	**p < 0.001**

For all proteins (mg/dl), except for ACT and SAA (µg/ml). Data presented as median and interquartile range (IQR)

*A2MG* alpha2-macroglobulin, *AAT* alpha1-antitrypsin, *ACT* alpha 1-antichymotrypsin, *AGP* alpha-1 acid glycoprotein, *ALB* albumin, *CP* ceruloplasmin, *hs-CRP* high sensitivity C-reactive protein, *HP* haptoglobin, *SAA* serum amyloid A. Two-sided p-values were calculated with Mann–Whitney-U test. A p-value below 0.05 was considered as a significant. The number of independent samples was n = 10 in each group

### Correlations between measured plasma APPs

In patients with COVID-19 strong positive correlations were found between plasma levels of hs-CRP and AAT (r = 0.7, p < 0.001), and between HP and AGP (r = 0.61, p < 0.01) (Table 4). When COVID-19 patients were segregated into subgroups, amongst the patients with a moderate disease, strong correlations were found between AAT and AGP (r = 0.91, p < 0.001), HP (r = 0.79, p < 0.01), hs-CRP (r = 0.72, p < 0.01), and CP (r = 0.89, p < 0.01). However, in severe COVID-19 patients the strongest positive correlations were found only between plasma levels of ACT and SAA, hs-CRP and AAT, and between CP and AAT, and negative correlations between HP and ACT as well as between HP and ALB.

**Table 4:** Pearson’s correlation of APP plasma levels in patients with COVID-19 disease (n = 20)

Protein	ACT	SAA	AGP	HP	AAT	hs-CRP	A2MG	CP	ALB
ACT	1	0.77***	− 0.19	− 0.47	− 0.16	− 0.20	0.23	0.12	0.45
SAA	0.77***	1	− 0.20	− 0.33	0.04	− 0.02	0.02	0.27	0.04
AGP	− 0.19	− 0.20	1	0.61**	0.67**	0.63**	0.03	0.49	− 0.41
HP	− 0.47	− 0.33	0.61**	1	0.52	0.57	− 0.26	0.20	− 0.51
AAT	− 0.16	0.04	0.67**	0.52	1	0.70***	− 0.13	0.72***	− 0.58
Hs-CRP	− 0.20	− 0.02	0.63**	0.57	0.70***	1	− 0.23	0.42	− 0.50
A2MG	0.23	0.02	0.03	− 0.26	− 013	− 0.23	1	0.17	0.49
CP	0.12	0.27	0.49	0.20	0.72***	0.42	0.17	1	− 0.31
ALB	0.45	0.04	− 0.41	− 0.51	− 0.58	− 0.50	0.49	− 0.31	1

*A2MG* alpha2-macroglobulin, *AAT* alpha1-antitrypsin, *ACT* alpha 1-antichymo-trypsin, *AGP* alpha-1 acid glycoprotein, *ALB* albumin, *CP* ceruloplasmin, *hs-CRP* high sensitivity C-reactive protein, *HP* haptoglobin, *SAA* serum amyloid A. Two-sided p-value < 0.05, two stars (**) indicate a two-sided p-value < 0.01, three stars (***) indicate a two-sided p-value < 0.001. A p-value < 0.05 is considered as significant

### Whole plasma N-glycan analysis

**Fig. 3:**
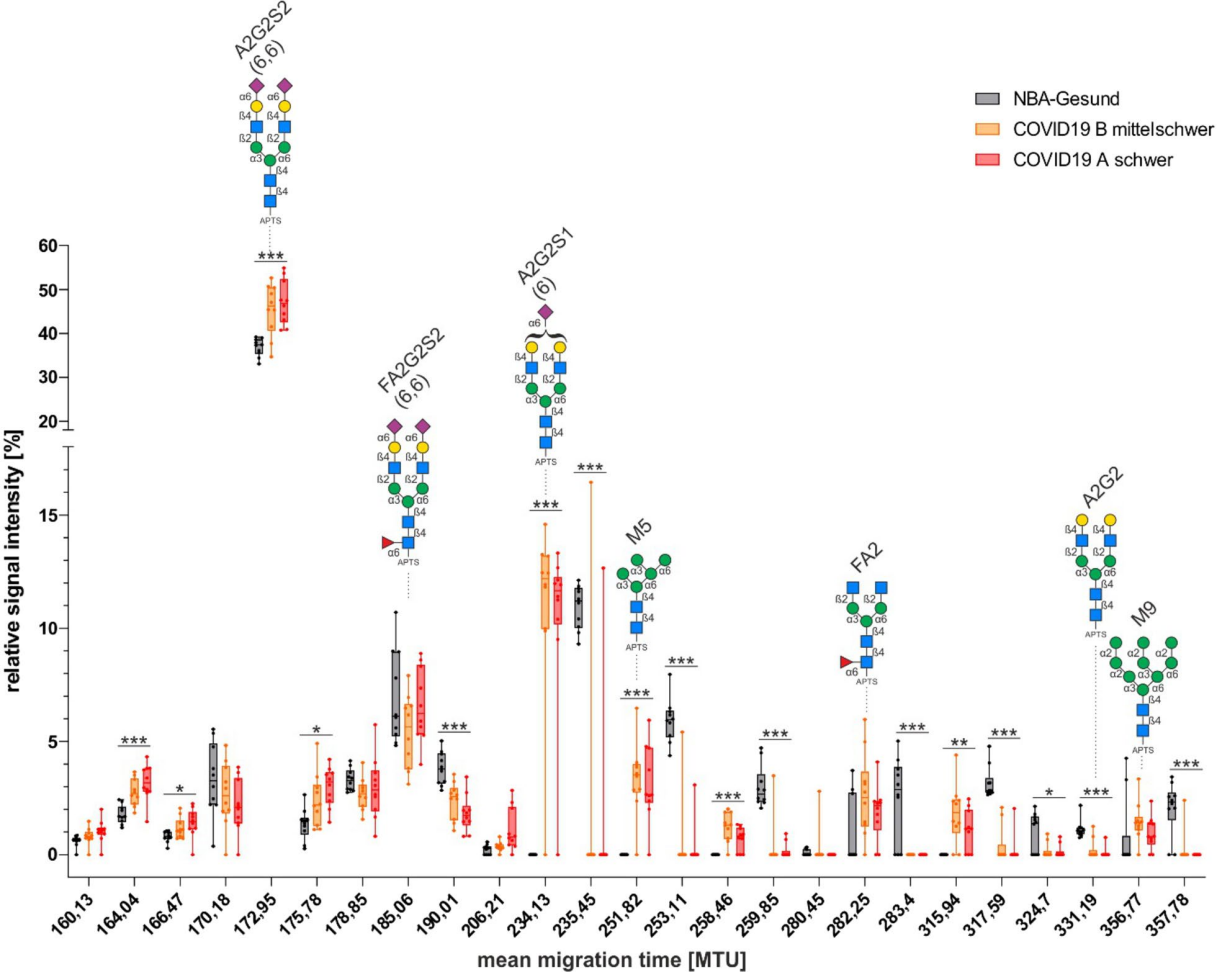
N-glycan profiling of whole serum proteins revealed changes in glycosylation state during COVID-19 infection. Box plot display relative signal intensities of whole-serum derived N-glycans of moderate COVID-19 patients, severe-COVID-19 patients, and healthy controls (n = 10, each). Assigned N-glycans are depicted as pictograms with purple diamond: sialic acid; yellow circle: galactose; blue square: N-acetylglucosamine; green circle: mannose, red triangle: fucose; blue circle: glucose. Symbolic representation of pictograms follows the guidelines of Symbol Nomenclature for Glycans (SNFG) [29]. Student’s t-test two-sided: one star (*) indicate a p-value < 0.05, two stars (**) indicate a p-value < 0.01, and three stars (***) indicate a p-value < 0.001 based on less significant differences when comparing control and each COVID-19 group

N-glycosylation of whole plasma proteins of COVID-19 patients (moderate and severe) and healthy controls were comparatively analyzed by capillary gel electrophoresis coupled to laser-induced fluorescence detection (CGE-LIF). Thereby, 25 structures, exceeding a threshold of 2% relative signal intensity, were identified (Fig. 3). Of these, for 17 N-glycans relative levels were significantly different between COVID-19 patients and healthy controls. Of note, relative levels of oligomannose (M5, M9) and di-antennary sialylated glycans (A2G2S2 (6,6), A2G2S1(6) were higher in SARS-CoV-2 infected patients relative to controls. On the other hand, the corresponding di-antennary non-sialylated glycan (A2G2) was significantly lower in COVID-19 patients. Relative levels of two core fucosylated N-glycans were comparable between all groups (FA2G2S2(6,6), FA2). Further peaks also displayed significantly different intensities between study groups; however, in our database these picks were not assigned to specific glycan structures. The differences between moderate and severe cases were less pronounced. Here we found only two significantly different structures, which unfortunately could not be annotated.

## Discussion

For unknown reasons some individuals infected with SARS-CoV-2 develop moderate symptoms and others become severely ill. Furthermore, patients differ in their response to therapies [30, 31]. These variable clinical manifestations reflect the host`s genetics, immunity, and comorbidities [32, 33]. Therefore, the discovery of biomarker signatures differentiating COVID-19 patients with different severities remains of importance. Since plasma/serum can be collected noninvasively, analyses of circulating protein levels and posttranslational modifications can give much information about the host`s proteome response to COVID-19 [34]. We conducted a pilot study in a small cohort of COVID-19 patients exhibiting severe or moderate symptoms to determine plasma levels of M30/M65, ADAMST13, various APPs and whole protein N-glycosylation profile. Although other studies have already investigated many proteins in COVID-19 patients, our findings may still contribute to the better understanding of the proteome responses to the infection. We provide results from a parallel measurement of nine plasma APPs concentrations, six of which were significantly higher in COVID-19 patients than in healthy subjects. Among these latter, median levels above reference values were found for AGP: 167.0 mg/dl (ref. 51–117 mg/dl), HP: 271.5 mg/dl (ref. 36–195 mg/dl), AAT: 235.5 mg/dl (ref. 88–174 mg/dl) and hs-CRP: 9.68 mg/dl (ref. less than 0.744 mg/dl). Albeit within the reference range, the levels of ALB were significantly lower, but CP were significantly higher in COVID-19 patients as compared to healthy controls.

Though changes in APP levels reflect general inflammatory response, altered magnitudes and profiles of these proteins might mirror distinct processes involved in the SARS-CoV-2 infection and disease progression. For instance, repeated studies show that levels of CRP are useful as a biomarker to follow COVID-19 progression [35], and patient management [36–38]. AAT is also acknowledged as one of the key immunomodulatory proteins during the SARS-CoV-2 infection [22, 39] beneficial for COVID-19 patients [40, 41]. AAT is a broad inhibitor of proteases and interacts with different pro-inflammatory substances [42]. Moreover, AAT inhibits the metalloproteinase domain 17 [43] and the type II transmembrane serine protease [39], two host proteases facilitating SARS-CoV-2 viral entry, replication and the pathogenesis of viral infections. AGP, which also was found to be higher in severe versus moderate COVID-19 patients, belongs to the lipocalin protein family. Functions of AGP remain incompletely understood, although typically associated with anti-inflammatory, immune modulatory, and sphingolipid metabolism [44, 45]. On the contrary, levels of A2MG and, especially ALB, decreased in association with COVID-19 severity. A2MG is one of the major blood proteins binding a wide range of substances, such as TGF-β1, TNF-α, IL-1β, and hormones, and inhibiting proteases, like trypsin, chymotrypsin, elastase, metalloproteinases as well as parasite-derived proteinases, and it is involved in blood coagulation and fibrinolysis [46]. Moreover, A2MG binds blood iron, zinc, and copper ions stronger than ALB, and acts as a serum copper transporter. The sequestering of metal ions by A2MG or ALB is one of the host defense strategies against infections [47]. Previous experimental studies have shown that the administration of A2MG prolongs graft survival and protects against sepsis [48, 49]. The impaired fibrinolysis together with lower levels of A2MG suggested as a risk of asthma exacerbations [50]. COVID-19 disease has not yet been associated with low plasma levels of A2MG, which inspires further investigations. However, low serum levels of ALB have already been associated with poor COVID-19 patient prognosis [51, 52]. Researchers also demonstrated a negative relationship between serum ALB levels and a risk of developing thromboembolism [53]. In some patients, however, increased hemolysis and altered levels of hemoglobin and heme-scavenging proteins (i.e., hemopexin, ALB or HP) were described [54]. Thus, triggered induction of AAT, AGP, CRP or other APPs as well as the reduction of A2MG and ALB during the SARS-CoV-2 infection may mirror specific pathological processes. However, it remains unknown which concentrations of these proteins are required to be protective and/or become detrimental.

N-glycosylation is a highly dynamic posttranslational modification strongly affected by viral infections and inflammation [55]. APPs represent a fraction of mostly glycosylated plasma proteins, and therefore, we anticipated changes in N-glycan levels upon SARS-CoV-2 infection. Our N-glycan analysis of whole plasma proteins revealed an increase in the di-antennary di-sialylated glycan A2G2S2(6,6) in COVID-19 patients, which is the most abundant N-glycan on AAT [56]. Increased relative levels of A2G2S2(6,6) could be explained by the observed higher plasma levels of AAT in COVID-19 patients. Also, the di-antennary mono-sialylated glycan A2G2S1(6) is highly elevated in the serum of COVID-19 patients whereas the corresponding non-sialylated di-antennary N-glycan A2G2 was even significantly decreased in COVID-19 patients. These findings hint towards an elevated sialylation of serum protein N-glycans upon SARS-CoV-2 infection. Accordingly, scientists reported that SARS-CoV-2 not only induces a production but also increases the sialylation of AAT [22]. This latter was linked to enhance anti-inflammatory properties of AAT [57]. Furthermore, we detected increased levels of oligomannose N-glycans (M5, M9) from serum proteins of COVID-19 patients. It is well known that glycoproteins with terminal mannoses are more rapidly cleared from the circulation than their complex glycosylated counterparts [58] which might therefore exert broad effects on serum levels of diverse glycoproteins including immunoglobulins [59].

Finally, there are increasing reports regarding cell damage and thromboembolism in COVID-19 cases. As COVID-19 is associated with hypercytokinaemia, cytokines, like IL-1, IL-6 and TNFα, may induce cell death. Therefore, we sought to quantify circulating levels of full length (M65) and caspase-cleaved (M30) cytokeratin 18 (CK-18), as markers of apoptotic and necrotic cell death. Indeed, M65 (total cell death) and M30 (apoptosis) were significantly elevated in COVID-19 patients compared with healthy controls. It is noteworthy that M30/M65 ratio did not differ between COVID-19 patients with different severities but was significantly lower than in controls. This latter suggests that cytotoxic or ischemic necrosis might be the predominant pathway of cell death in these patients. In line, other studies suggested that increased cell death in COVID-19 patients relates to defective organ perfusion, particularly in those who develop microvascular thrombosis [60]. We also found significantly lower levels of ADAMTS13 in COVID-19 patients relative to controls. The reciprocal relationship between VWF and ADAMTS13 in thrombosis is widely studied [61], and several groups reported that COVID-19 causes a significant increase in formation of large VWF multimers, and decrease in activity and/or levels of ADAMTS13 [62–64].

## Conclusions

The number of patients and controls analyzed in this study is too small to allow for controlling potential confounding factors such as age, sex, smoking status, comorbidities, and therapies. The correlations between identified protein levels and changes in glycosylation need further investigations in larger cohorts. Nevertheless, our findings support a notion that inflammatory markers of different pathways when monitored simultaneously and combined with clinical data may help to improve prognosis and outcomes for COVID-19 patients.

## Data Availability

The data that support the findings of this study are available from the corresponding author, SJ, upon reasonable request.

## Abbreviations

A2MG: Alpha2-macroglobulin
AAT: Alpha1-antitrypsin
ACT: Alpha 1-antichymo-trypsin
AGP: Alpha-1 acid glycoprotein
ADAMTS13: A disintegrin-like and metalloprotease with thrombospondin type-1 motif 13
ALB: Albumin
APP: Acute phase protein
CP: Ceruloplasmin
ECMO: Extracorporeal membrane oxygenation
GOLD: Global initiative for chronic Obstructive Lung Disease
hs-CRP: High sensitivity C-reactive protein
HP: Haptoglobin
ICU: Intensive care unit
SAA: Serum amyloid A
SpO_2_: Peripheral oxygen saturation
